# Structure elucidation and proposed *de novo* synthesis of an unusual mono-rhamnolipid by *Pseudomonas guguanensis* from Chennai Port area

**DOI:** 10.1038/s41598-019-42045-9

**Published:** 2019-04-12

**Authors:** K. C. Ramya Devi, R. Lakshmi Sundaram, Sivamurugan Vajiravelu, Vidya Vasudevan, Gnanambal K. Mary Elizabeth

**Affiliations:** 1 Department of Biotechnology, SRI RAMACHANDRA Institute of Higher Education and Research, Deemed to be University (DU), Porur, Chennai, 600 116 Tamil Nadu India; 2 Central Research Facility (CRF), SRI RAMACHANDRA Institute of Higher Education and Research, Deemed to be University (DU), Porur, Chennai, 600 116 Tamil Nadu India; 3 PG & Research Department of Chemistry, Pachaiyappa’s College, Chennai, 600 030 Tamil Nadu India

## Abstract

In this paper, we describe the isolation of an unusual type of high molecular weight monorhamnolipid attached to esters of palmitic, stearic, hexa and octadecanoic acids as against the routinely reported di-rhamnolipids linked to hydroxydecanoic acids. The bioemulsifier was column-purified and the chemical nature of the compound was elucidated using FT-IR, GC-MS and 1D [^1^H and^13^C] and 2D NMR. This monorhamnolipid is extracted from a bacterium, *Pseudomonas guganensis* and is not reported to have biological activities, let alone emulsification abilities. The bacterium continually produced rhamnolipids when nourished with *n*-hexadecane as its lone carbon source. The extracellularly secreted monorhamnolipids are capable of degrading hydrocarbons, with most preference to *n*-hexadecane [EI_24_ of 56 ± 1.42% by 2 mL of the spent medium]. Whilst the crude ethyl acetate partitioned extract had an EI_24_ of 65 ± 1.43%; the purified rhamnolipid product showed 78 ± 1.75% both at 12.5 mg/mL concentration. The used-up *n-*hexadecane is biotransformed to prepare its own rhamnolipids which in return is utilized to degrade *n*-alkanes thus creating a circular pathway which is proposed herein. This bacterium can be seen as a new source of bioemulsifier to reduce hydrocarbon in polluted waters.

## Introduction

Long-chained aliphatic hydrocarbons, though described as robust substrates are still utilized only by specific bacteria, which are known to show up soon after an oil spill, while the non-hydrocarbon utilizing groups diminish^1^. In an aquatic environment with chronic hydrocarbon contamination, an increased oxidation rate of *n*-hexadecane will be observed^2–4^ indicating its uptake. *n*-Hexadecane is usually represented as a significant component of crude oil in aquatic environments, owing to its less volatility which is attributed to more carbon atoms (C-16) and hence is a routinely utilized carbon source by marine bacteria prevalent in that locality. Right from 1970s, there are researchers, who continually report significant bacterial populations to have a preferred carbon source as *n*-hexadecane which is particularly mineralized by the secretion of bioemulsifiers^5–7^.

It has been demonstrated all over these years that rhamnolipids, a well-known class of bioemulsifier is produced and secreted profusely by bacteria to utilize *n*-alkanes of higher molecular weights (carbon atoms >14), whose dispersion would otherwise be cumbersome because of their high log_*P*_ values^8–10^. Hence there is a high focus on the production of rhamnolipids of various types from microorganisms specifically to degrade dense Non-Aqueous-Phase Liquids [NAPLs] like, *n*-hexadecane. With this notion, the present investigation describes the isolation of an unusual type of monorhamnolipid for the first time from *Pseudomonas guganensis* and evaluate its emulsification of *n*-hexadecane in water. The paper also explains in detail the structural characteristics of three compounds which collectively contributed to *n-*hexadecane degradation and subsequent utilization, with the support of necessary spectral data. The spectral data were also used to construct a plausible pathway for the *de novo* synthesis of bioemulsifier.

## Materials and Methods

### Microbiological sampling of waters from various locations and initial screening of bacteria with hydrocarbon degrading capabilities

A series of bacterial isolations were done [using peptone and yeast extract containing media with different mineral salt supplementations in double filtered seawater] for water samples various locations of Southeast coast of India for screening hydrocarbon degrading efficiencies. The sampling sites included, Tuticorin (8.76° N, 78.13° E), Olaikuda boat jetty (9.28° N, 79.31° E), jetty for fishing boats of Gulf of Mannar (18.37° N, 83.99° E) and three points of Chennai harbor (13.0815° N, 80.2921° E), ie., 1. Signal station, 2. Container area and 3. Sailing area. A total of 849 bacteria were isolated and all of the isolates were tested for hydrocarbon emulsification abilities. Formation of a visible hydrocarbon emulsion in water was scored as a positive result for the investigation, which is the standard test to evaluate the efficacy of an emulsifier. For that, a loopful of luxuriantly grown isolate was transferred to a 10 mL ZoBell Marine Broth (ZMB) [contains 5% peptone and 1% yeast extract in double filtered seawater] and orbital shaken at 150 rpm at 30 °C overnight. After that, 5 mL of the grown cultures (OD_600_ − 1) were used for obtaining Cell-Free Supernatants (CFSs) by spinning down at 8000 rpm (4 °C) for 30 min and used for the assay.

The Emulsification Index_24_ (EI_24_) of all the isolates was assayed following the methodologies of^11^. Accordingly, 2 mL of diesel/kerosene/*n*-hexadecane (Sigma) were added in equal volumes to each of the CFSs and homogenized in a vortex at high speed for 2 min. The mixture was allowed to stand and the stability of the emulsions was measured after 24 h and then emulsification indices were calculated using the formula: EI_24_% = [total height of the emulsified layer/total height of the liquid column] * 100. Out of the 849 isolates screened for the purpose, it was only two of them that passed these investigations and hence these two isolates [assigned as A and B] were taken for further examination.

### Classification of potent hydrocarbon emulsifying isolates A and B by ribotyping

Selected isolates were ribotyped using 16S rRNA gene sequencing and DNA extraction was done based on the methodologies developed by Birnboim and Doly (1979), incorporating minor changes. The 16S rRNA coding gene was amplified in a thermocycler (G-STROM, Somerset, UK) using specific primers [27 F- AGAGTTTGATCCTGGCTCAG, 1492 R – GGTACCTTGTTACGACTT]^12^. Reaction volumes of 10 µL [10 pmol of each of the primers, 10 µmol dNTPs, 1U Taq polymerase and 50 ng/µL of that bacterial genomic DNA] were prepared. PCR program was followed thus: (1) initial denaturation of the template at 95 °C for 5 mins; (ii) denaturation at 95 °C for 30 s, (iii) annealing at 55. 4 °C for 30 s and (iv) an extension cycle at 72 °C for 30 s with an additional final extension at 72 °C for 5 min. The processes were in repetition for 30 cycles and amplicons were loaded on a 2% agarose gel for separation and visualization and thereafter submitted to ABI 3730 DNA Analyzer (ThermoFisher, USA) for sequence determination^13^. The sequences were compared with the known ones in the National Centre for Biological Information (NCBI) database using Basic Local Alignment Search Tool (BLAST version 2.2.12) and a phylogenetic tree was thus constructed (MEGA 6 software). In parallel, the isolates were also submitted for identification at the Institute of Microbial Technology (IMTECH), Chandigarh for confirmation. To our ill fortune, isolate A turned out to be the recognized bioemulsifier producer, *Pseudomonas aeruginosa*, whilst B was identified as *Pseudomonas guganensis*, which is isolated by us for the first time in the Indian waters [GenBank accession number: KU302611]. The latter has never been tested for any bioactivities, leave alone emulsification efficiency since its discovery in 2013 from the hot springs of Taiwan till date. Therefore, we patented the process of extraction of unusual monorhamnolipids from this bacterium in India [Application No: 201641037265 A; Date of publication: 17/11/2017]

### Partitioning protocols, crude bioemulsifier preparations, evaluation of EI_24_ and gram-scale production of the crude material

This section details the procedures for the mass production of bioemulsifiers from *P*. *guguanensis* and we set *P*. *aeruginosa* as a positive control for preliminary assays. For extraction of crude bioemulsifiers from *P*. *guguanensis*, one loop of the lush grown isolate was introduced into 10 mL of ZMB and orbital shaken at 150 rpm at 30 °C overnight in the same manner as performed for the preliminary screening experiments. After that 5 mL of the grown culture (OD_660_ − 1) was introduced into 1 L half strength ZMB for a period of 7 days. Subsequently the CFSs were obtained by spinning at 8000 rpm at 4 °C for 30 mins. The supernatant was briefly acidified to pH 2.0 using 1N HCl and kept overnight at 4 °C. Further to this, actual extraction procedure involved mixing of spent broth-to-solvents in equal volumes in a separating funnel which was then swiftly shaken for a minute and allowed to stand for an hour for phase separations. The solvents used for partitioning were in this order: 1. hexane, 2. dichloromethane, 3. ethyl acetate and 4. methanol. Organic phases were separated from the aqueous ones and the procedures were repeated thrice and the pooled organic phases from each of the partitions were concentrated by vacuum distillation. Partitioning and extraction procedures were adopted from the methodologies listed by^14^. Finally, the crude bioemulsifier yield from each of those solvents was noted and the completely dehydrated material was rechecked for emulsification properties and stored in amber-colored bottles at 4 °C until next usage.

At this stage, when only the ethyl acetate partition of *P*. *guguanensis* was able to show appreciable emulsification to *n-*hexadecane [for diesel and kerosene- data not shown here], further investigations were done only for that sequential extract. For the sake of purification of the emulsifier, extracted material in gram quantities was required. Therefore, mass cultivation of the isolate was performed using 40 L half strength ZMB by employing optimal growth conditions and extraction procedures and the crude bioemulsifier was then dried and weighed. Extraction procedures were repeated until there was no more extractable biomeulsifer and thus the mass production of crude bioemulsifier was achieved to the tune of 2 g in a laboratory scale which sufficed the demand of the raw material for further purification on a silica gel column.

### Column chromatography conditions and separation of active compounds

As a prelude to doing column chromatography of the active crude extracts, Thin Layer Chromatograms (TLCs) (Merck, India) were obtained using the elution systems: (i) hexane: ethyl acetate; (ii) ethyl acetate: methanol in different ratios out of which ethyl acetate: methanol [5:5] showed better resolution. TLCs were viewed in UV (254 and 366 nm), white light and derivatized using 10% methanol in sulfuric acid (v/v). To purify the crude bioemulsifer, 2 g of the active ethyl acetate extract was subjected to column chromatography using silica gel [mesh size 230–400] as the packing material^15^ and eluents, from hexane to methanol through ethyl acetate were used in this order: hexane; 90% hexane: 10% ethyl acetate; 70% hexane: 30% ethyl acetate; 50% hexane: 50% ethyl acetate; 30% hexane: 70% ethyl acetate; 10% hexane: 90% ethyl acetate; ethyl acetate; 97.5% ethyl acetate: 2.5% methanol; 95% ethyl acetate: 5% methanol; 92.5% ethyl acetate: 7.5% methanol; 90% ethyl acetate: 10% methanol; 80% ethyl acetate: 20% methanol; 70% ethyl acetate: 30% methanol; 60% ethyl acetate: 40% methanol; 50% ethyl acetate: 50% methanol; 40% ethyl acetate: 60% methanol; 30% ethyl acetate: 70% methanol; 20% ethyl acetate: 80% methanol; 10% ethyl acetate: 90% methanol and methanol. Elutions were performed at room temperature and all the fractions were collected, dried and checked for EI_24_ (Cooper and Goldenberg, 1987).

### Separation of active fraction using Preparative Thin Layer Chromatography (*p*-TLC) and structural investigation of the active compounds

The active fraction had closely resolved 3 compounds, and hence a *p*-TLC was found to be necessary for further separation and purification of individual compounds. The three compounds assigned as 1, 2 and 3 were successfully separated and whether or not (1) these three compounds together or (2) in combinations of two or (3) in isolation gave maximum emulsification was felt necessary to be determined. Based on EI_24_ assay, it was confirmed that all the three compounds only when present together contributed to emulsification and thereafter we went on to investigate the structural and chemical characteristics of each of them. Accordingly, 5 mg of individual compounds 1, 2 and 3 were compressed with 200 mg of KBr to form pellets and submitted to FT-IR (Jasco 4000 series, Maryland, USA). For GC-MS studies, samples were vigorously dissolved in 1 mL of methanol, filtered in a nylon filter (0.25 µ) and from this 1 µL of the sample was injected into a Triple Quadrupoles, TSQ quantum GC-MS (Thermo Scientific Massachusetts, USA) column: 30 mm × 0.25 mm ID × 1 μm, composed of 5% phenyl and 95% methyl polysiloxane [Column conditions: Electron impact mode at 70 eV; carrier gas: Helium; flow rate: 1 mL/min. Injector temperature: 290 °C; fragments size: 45 to 450 Da; Total running time: 36 min]. Similarly, for NMR analyses, the purified compounds were dissolved in deuterated DMSO d_6_ and fed to Nuclear Magnetic Resonance (NMR) spectrophotometer [Model: Bruker 400 MHz (Avance III)] for both ^1^H and ^13^C analyses. Two-dimensional NMR for each of the compounds were also recorded in the same machine, which included, Correlation Spectroscopy (COSY), Heteronuclear Single-Quantum Correlation Spectroscopy (HSQC), Heteronuclear Multiple-Bond Correlation Spectroscopy (HMBC) and Distortionless Enhancement by Polarization Transfer (DEPT-135) Spectroscopy.

## Results

### Isolation and growth conditions of bacteria from various locations and evaluation of emulsification capabilities

Out of an exhaustive sampling from six different areas across the Tamil Nadu coast, we were able to acquire 849 bacterial isolates which were individually tested for hydrocarbon emulsifying properties in water. EI_24_ was calculated for all of the isolates using standard protocols listed in Materials and Methods section. This vast screening program, to our utmost disbelief, yet realistic, yielded only two isolates (0.23%) exhibiting emulsification capabilities (S1). The strains were designated as A (EI_24_ - *n*-hexadecane [46 ± 2.12%], diesel [40 ± 1.41%] and kerosene [22 ± 1.40%]) and B (EI_24_ - *n*-hexadecane [56 ± 1.42%], diesel [46 ± 2.82%] and kerosene [48 ± 1.40%]). These values are obtained for 2 mL of the spent broth at Abs_660_ ~ 1 OD and the results are supplemented as S2. The strains were customarily cultured on Bushnell and Haas medium supplemented with increasing concentrations of diesel and *n*-hexadecane [0.5% increment for every 6 months] to achieve maximal acclimatization to that hydrocarbon source. In consequence, isolate B grows luxuriantly at 5% *n*-hexadecane supplementation [to the minimally enriched culture medium] after 45 months’ adaptation program and the strain is cultivated till date with still higher measures of *n*-hexadecane.

### Molecular identification of bacteria and gram-scale extraction of bioemulsifiers from CFSs

**Figure 1:**
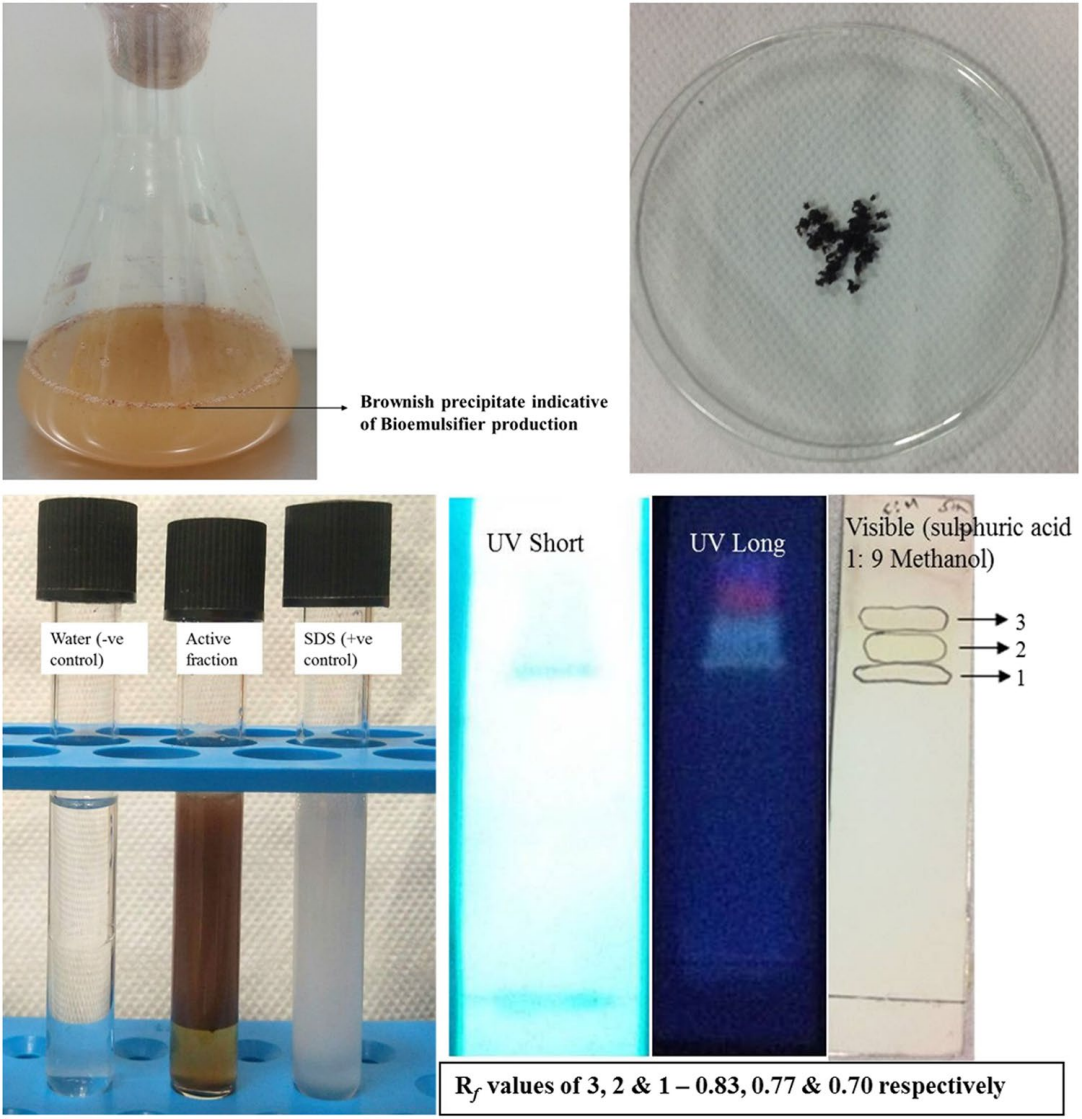
Laboratory-scale production of bioemulsifer by *P. guguanensis* [left] and dehydrated crude extract [right] in the top. Emulsification activity [left] and TLC pattern [run in EA:MeOH 5:5] of active fraction [right] at the bottom.

The strains were identified by 16S rRNA ribotyping as *Pseudomonas aeruginosa* (A) and *Pseudomonas guguanensis* (B) (gene sequences supplemented as S3). It is a broadly known fact that *P*. *aeruginosa* is a prolific producer of emulsifier and untiring efforts have been centered to this strain for evaluating its oil-degrading capabilities. Hence our focus was shifted to *P*. *guguanensis* which was encountered by us for the first time in Indian coastal waters, after its discovery in Taiwan in 2013. Subsequently for our preliminary experiments the wild-type *P*. *aeruginosa* that we isolated as kept as positive control together with a chemical surfactant, Sodium Dodecyl Sulfate (SDS). The used-up medium of the inoculants which was assumed to contain bioemulsifier was partitioned using solvents of different polarities. For that reason, a 1 L measure of the broth was added with equal volumes of solvents one after the other in the following order (hexane, dichloromethane, ethyl acetate and methanol), filtered separately and the crude extracts were concentrated. The extract yield was found to be highest for ethyl acetate (76 mg/L) and the least for methanol (53 mg/L). The crude emulsifier extracts from all the solvents were put to emulsification test using *n*-hexadecane and the highest EI_24_ was exhibited by the ethyl acetate extracted compounds (65 ± 1.43%) [Fig. 1] and the least for methanol (30 ± 0.87%), both at 12.5 mg/mL concentration (data not shown).

Therefore, ethyl acetate was used as the choice of solvent for maximal extraction of active emulsifier produced by large-scale cultivation of *P*. *guguanensis* after a seven-day incubation regimen. Cultures with Abs_660_ ~ 1 were mass-extracted from 40 L fermentation broth to yield 2 g of the active emulsifier. A small portion of this crude extract was performed with a quick EI_24_ and the property was re-confirmed.

### Column chromatography and purification of emulsifier

2 g of crude emulsifier was column-chromatographed using a sequence of mobile systems listed precisely in the Materials and Methods section and followed in that order. Close to 100 fractions were eluted and each of these was meticulously separated using TLC and reconfirmed for emulsification efficiencies. Fractions (9–18) eluted using 100% ethyl acetate were classified as active emulsifiers with an EI_24_: 78 ± 1.75% at 12.5 mg/mL concentration and all of these had the following things in common: (i) mobility on TLC; (ii) absorbance patterns in UV [254 and 366 nm], visible light and (iii) derivatizations and thus were pooled together and concentrated to dryness. Consequently, we were able to obtain 102 mg of an active fraction as a yellowish brown oily substance from the 2 g of the crude material (compound yield 0.051%). The active fraction had the following characteristics: Absorbencies at UV- both 254 and 366 nm, visible light and derivatizable using 10% methanol in sulphuric acid (v/v). The active fraction (EI_24_: 54 ± 1.07%) was however viewed to contain 3 spots with R_*f*_ values: 0.83, 0.77 & 0.70 and a further purification was felt required. A Preparative Thin Layer Chromatography (*p*TLC) was therefore carried out and all the three compounds were ably separated, purified and structurally determined using FT-IR, GC-MS and NMR 1D (^1^H and ^13^C) and 2D and the results are exhaustively described below.

### Chemical investigation of the compounds

#### Complete spectral study of compound **1**

**Figure 2:**
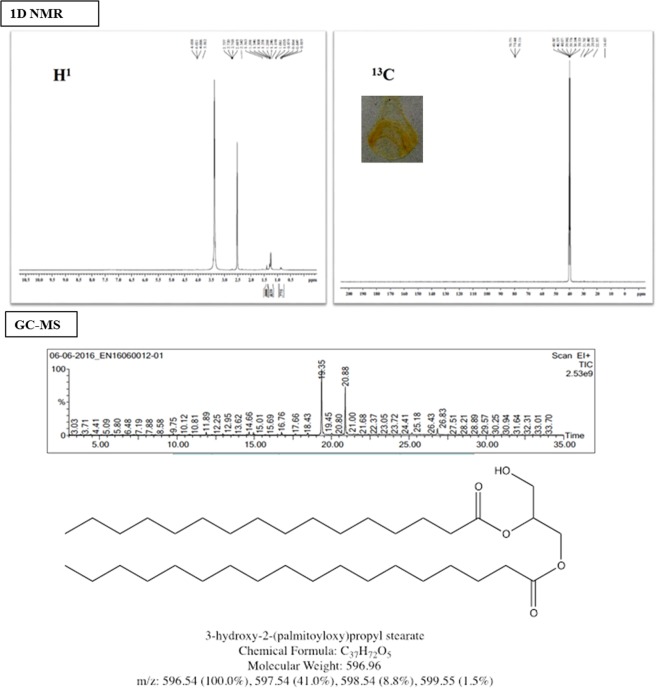
NMR spectroscopies [^1^H and ^13^C] and GC-MS fragmentation patterns of compound **1**.

Compound **1** was a dark yellowish oily precipitate that weighed 24 mg. FT-IR spectral data reveals the presence of alcoholic hydroxyl (−OH) group as broad peak in the region 3550–3450 cm^−1^ and indicates that hydroxyl groups were involved in the intramolecular hydrogen bonding. The compound showed very strong C-H stretching vibration at 2923 cm^−1^ to support the presence of long alkyl chains. The aliphatic ester carbonyl C=O and C-O stretching vibration were observed at 1743 and 1022 cm^−1^ respectively and compound **1**showed clean FT-IR vibration peaks to support the proposed structure. Gas Chromatography-Mass Spectrometry analysis of this compound showed two major peaks in the ratio of 60:40 at retention times, 19.35 and 20.88 min. The peak specifically at 19.35 min showed a fragmentation pattern of a glycerol ester of palmitic acid. The structure was substantiated by the existence of a molecular ion (M-OH), palmitic acid, palmitoyl cation and *n*-pentadecenyl cation which showed prominent peaks at m/z 313, 256, 239 and 209 respectively. Further, the occurrence of alkyl, ester carbonyl and hydroxyl methyl group is confirmed using ^1^H and ^13^C NMR spectroscopies. Not only that, a distinct peak at 20.88 min revealed that the compound contained a glycerol ester of stearic acid. The presence of glycerol ester of stearic acid is additionally confirmed by a molecular ion peak (M-OH) with m/z = 341, which is attributed to the loss of hydroxyl ion from the glycerol unit. Adding up, we also confirm the presence of stearic acid cation, stearyl cation and *n*-heptadecenyl cation with the peaks appearing at m/z 283, 267 and 239 respectively. Also, the presence of alkyl and ester group in the compound was confirmed by the peaks in ^1^H and ^13^C NMR spectral analyses. By corollary, compound **1** is assigned as 3 hydroxy-2-(palmytoyloxy) propyl stearate [C_37_H_72_O_5_] with a molecular weight of 596.96 Da and the spectral data are given as Fig. 2. The corresponding FT-IR and GC-MS patterns of this compound are given as S5 and 2D NMR spectroscopies as S6.

#### Complete spectral investigation of compound **2**

**Figure 3:**
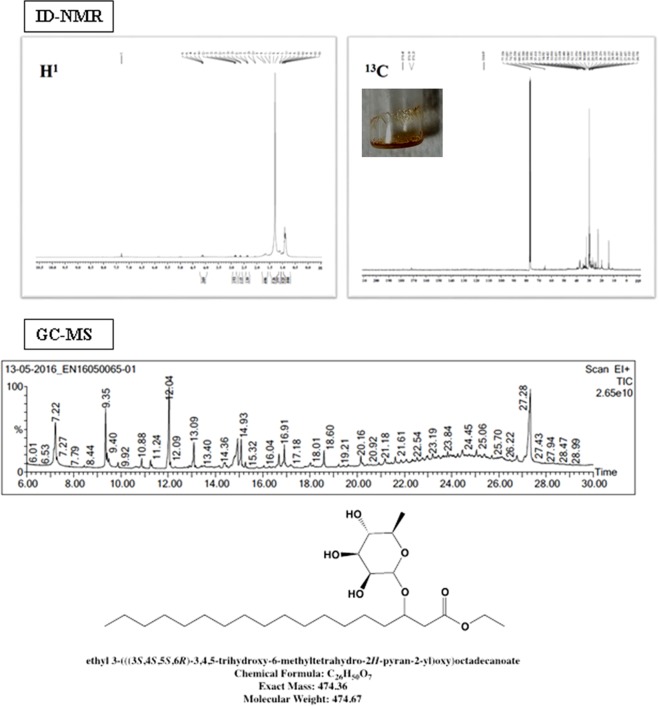
NMR spectroscopies [^1^H and ^13^C] and GC-MS fragmentation patterns of compound **2**.

Compound **2** appeared as glassy white easily breakable fragile crystals (7 mm) with yellow oily substance, which represents rhamnose sugars attached to lipids (41 mg). The presence of multiple alcoholic hydroxyl groups (-OH) observed as sharp and broad peaks in the region 3644–3475 cm^−1^ in FT-IR readout supports that these alcoholic groups were involved in inter as well as intramolecular hydrogen bonding. The aliphatic C-H stretching vibration is shown at 2923 and 2854 cm^−1^ and the presence of ester carbonyl (-C=O and C-O) is supported from its stretching vibration at 1739 and 1018 cm^−1^ respectively. GC-MS of this compound showed major peaks at 7.22, 9.35 and 27.28 min and the corresponding mass spectrum was recorded. Accordingly, a 7.22 min molecular ion peak at m/z 429 (M-OH), with a loss of m/z = 17 for a hydroxyl group has been assigned to the ethyl ester of 3-hydroxy palmitic acid containing rhamnose sugar unit. The presence of rhamnose sugar units was re-confirmed by the presence mass peak at m/z 147. Interestingly, the base peak at m/z 341 appeared after losing m/z = 88, thereby revealing the cleavage at C2-C3 attached to -O-rhamnose and an ester, C=O Presence of ester carbonyl, carbon connected OH and C=O was substantiated by ^13^C NMR and that of methylene and rhamnose protons using ^1^H NMR and all the spectral results are given as Fig. 3.

At 9.35 min, GC-MS showed a peak at m/z = 371 (M-3) which has been assigned to ethyl ester of 3-hydroxy octadecanoic acid containing rhamnose subunits attached to C3-hydroxyl group. Furthermore, the above interpretation was supported by the presence of two mass peaks each at m/z 327 and 147 corresponding to ethyl ester of 3-hydroxy octadecanoic acid and rhamnose units, which suggests that the cleavage had to happen at C-O bond connecting rhamnose and fatty acid units. In addition to the above evidence, the loss of ethoxy group (m/z = 45) from the fatty acid showed a mass peak at m/z 281, which corresponds to acyl cation of 3-hydroxy octadecanoic acid. ^1^H and ^13^C NMR spectrum of the compound too confirmed the presence of ester carbonyl and alkyl protons.

Further, a clear molecular ion peak at m/z 329 (M + 1), especially at 27.34 min, is apparently indicative of an ethyl ester of 3-hydroxy octadecanoic acid. The mass peak at m/z 243 corresponds to the loss of mass m/z 88 due to the cleavage between C2-C3 attached to hydroxyl and ester carbonyl groups. Hence compound 2, based on GC-MS and NMR spectroscopies is identified to be an ethyl 3-(((3*S*,4*S*,5*S*,6*R*)-3,4,5-trihydroxy-6-methyl tetrahydro-2H-pyran-2-yl) oxy)octadecanoate [C_26_H_50_O_7_] with a molecular weight of 474.67 Da. The corresponding FT-IR, GC-MS patterns of this compound are given as S7 and the 2D NMR spectroscopies as S8.

#### Complete spectral interpretation of compound **3**

**Figure 4:**
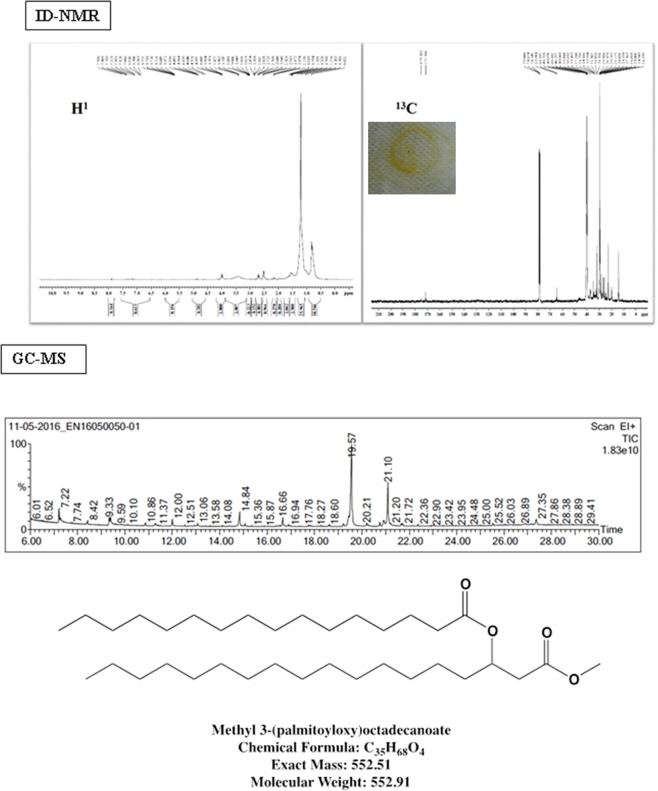
NMR spectroscopies [^1^H and ^13^C] and GC-MS fragmentation patterns of compound **3**.

Compound** 3** was separated as a dark yellowish brown oily substance (28 mg). Presence of intramolecularly bound alcoholic hydroxyl (-OH) groups is greatly supported by the broad peak that appeared in the region between 3550 and 3400 cm^−1^ in FT-IR spectral datum. The stretching vibration of C-H bonds present in the alkyl chain appeared at 2923 and 2854 cm^−1^ and that of ester carbonyl C=O and C-O at 1739 and 1022 cm^−1^ respectively. The compound showed two distinct peaks on GC-MS with retention times: 19.57 and 21.10 min. GC-MS peak at 19.35 min showed fragmentation pattern similar to that of palmitic acid. The structure was then confirmed by the presence of a palmitic acid, palmitoyl cation and *n*-pentadecenyl cation which showed characteristic peaks at m/z = 256, 239 and 207 respectively. Another peak appeared at 21.10 min that possessed a spectrum similar to those of methyl esters of 3-hydroxy hexadecanoic acid, which corresponds to the mass, m/z = 285 (M-3). Added to this, the fragmentations due to the loss of hydroxyl group showed a peak at m/z 267 that associates the existence of a methyl ester of palmitic acid. Further, the incidence of alkyl, ester carbonyl and alkyl groups attached to the hydroxyl and methylene attached carbonyl ones were confirmed using ^1^H NMR and ^13^C NMR. Also the occurrence of two carbonyl peaks in ^13^C NMR spectrum relates to the possibility of the presence of more than one ester carbonyl group. The compound was thus identified as methyl 3-(palmitoyloxy) octadecanoate [C_35_H_68_O_4_] with a molecular weight of 552.91 Da. We had observed that this compound is unstable under GC conditions and it is suspected that it may also decompose to give two additional peaks in GC. All the GC-MS data are provided as Fig. 4 and the corresponding FT-IR spectroscopy as S9.

## Discussion

We spotted along with well-known *Pseudomonas aeruginosa*, barely known species of this genus, i.e., *guguanensis*, from the oil-filled container area of Chennai Port of the Tamil Nadu coastal belt of South India for the first time. It is astounding to mention that only these two isolates out of the 849 had the capacities to disperse *n*-hexadecane, diesel, and kerosene as micro-emulsions in water. It is perhaps true for two reasons: (i) most of the heterotrophic non-differentiating marine bacteria those survive in oligotrophic conditions prevail in a state of dormancy/starvation^16–18^ (ii) uptake and metabolism of substrates like hydrocarbons is tough, especially when the organisms are obliged to use those as the solitary source of carbon when deprived of all other nutrients^19,20^. Not only that, extracellular secretion of emulsifiers to increase the surface area of the substrates, translocation of dispersed hydrocarbon across the cell membrane and further utilization, all demand much energy. All these facts put together, clearly explain the lesser recoverability of hydrocarbon using bacteria from nature as is the case with the present study. *Pseudomonas guganensis* grows luxuriantly after acclimatization for a period of 45 months with 5% *n*-hexadecane supplemented to minimal nutrient medium [Bushnell and Haas medium], which is the routinely suggested medium for recovering oil-degrading bacteria^21^. The medium contains only mineral salts with *n*-hexadecane as the sole source of carbon and the isolate was cultivated in 40 L of broth for the mass production of metabolites. It is noteworthy to mention that during exponential/stationary phases [between the 4^th^ and 7^th^ day], the ones which formed dark yellow/brown colored patches along the walls on Erlenmeyer flasks invariably produced bioemulsifiers of this type. We could thus ensure, even ahead of extraction procedures, that an absence of dark patch confirms lesser/no production of this kind of bioemulsifier, which we discovered during optimization studies using Response Surface Methodology (RSM) [data not shown]. The extract yield was found to be the highest for ethyl acetate (76 mg/L) and the least for methanol (53 mg/L). We were able to observe that the ethyl acetate extractable metabolites not only had the highest yield but also enhanced activity [EI_24_: 65 ± 1.43% at 12.5 mg/mL] than other solvents thus establishing a positive correlation for both the parameters. Crude ethyl acetate extract was therefore subjected to normal silica gel chromatography and fractions 9–18, which possessed emulsification [(EI_24_: 78 ± 1.75%) at 12.5 mg/mL concentration] were noted to have three compounds within. All the three compounds were *p*-TLC purified; nevertheless, we wanted to probe whether or not these compounds singly or in combinations could exhibit emulsification. Accordingly, neither the individual compounds, 1/2/3 nor when used in a combination of two [1 with 2/2 with 3/1 with 3], were able to exhibit emulsification. Therefore, we conclude that all the 3 compounds synergistically contribute to the emulsification activity. Also the bioemulsifier was found to be of high molecular weight with a total mass of 1264.52 daltons for all the three compounds taken together.

*P*. *guguanensis*, encountered in the present study was initially discovered in the hot springs of Guguan (24° 12′ 13.46′′ N 121° 00′ 29.49′′ E, 711 m), Taiwan^22^ and after that we encountered the strain in the oil-spread waters of Chennai Harbor. After its discovery there has been not a single research article on this strain, leave alone bioactivity/ecological roles which could be perhaps due to the less prevalence of this organism. The bacterium is highly aerobic, Gram-stain-negative bacillus with a polar flagellum for motility and is known to tolerate salinities up to 7%. The isolate formed light yellow colored colonies in ZoBell Marine Agar/Bushnell and Haas Agar.

With regard to the biosynthesis of rhamnolipid, as illustrated in the Results section of this paper, *P*. *guguanensis* produced only monorhamnose, which is in some divergence with previous studies, where researchers routinely encounter di over monorhamnolipids. Generally speaking, occurrence of dirhamnose units linked to β-hydroxydecanoate/α-decenoic acids is the most prevalent type of bioemulsifier by and large reported from Pseudomonads, when *n*-hexadecane^8,23^ or for that instance any *n*-alkanes^24,25^ are used as lone source of carbon. Whatever be the case, the predominance of the type of rhamnose (mono/di) is dictated by few variables, inclusive of the type of carbon source and the producer strain^26–28^. On the other hand, the lipid portion of the emulsifier had an ester of palmitic acid derivative, hexadecanoic acid. The plausible explanation of the production of palmitates is only by the oxidation of *n*-hexadecane supplied to the medium, which could otherwise not be possible with the minimal nutrition provided by Bushnell and Haas medium. The theory thus is confirmed by the survival of the bacterium in oxygen-rich conditions (continuous orbital shaking for 7 days), which could have favored high oxidation rates to biotransform *n*-hexadecane to palmitates. There have been many reports to precisely confirm rapid oxidation of a range of alkanes, specifically *n-*hexadecane by various species of Pseudomonads to esters of palmitic acid^29–31^. Medium chain fatty acids like octanoate, laurate, myristate are the most amenable substrates for biotransformation after uptake, whereas *n*-hexadecane/stearate are oxidized at much slower rates which explains the production of emulsifiers by *P*. *guganensis* at late stationary phases.

When the spent broth of *P*. *guguanensis* can exhibit EI_24_: 56 ± 1.42% [2 mL drawn of Abs_660_ ~ 1 OD culture], its extracted emulsifier from ethyl acetate can display 65 ± 1.43%. The purified rhamnolipids were able to showcase emulsification of up to 78 ± 1.75% as against the positive control SDS [EI_24_: 91 ± 0.5%] all at 12.5 mg/mL concentration and all these results are supplied as S4. At the same time, when *n*-hexadecane is supplied to the cultures [Abs_660_ ~ 1.76 OD], 84.3% removal was noticed on day 7 ie., 111.38 mg/mL out of 132 mg/mL of *n*-hexadecane supplied was removed [as confirmed by us during previous optimization studies for which the datum is not shown here]. This explains that degradation and simultaneous utilization of the broken products of *n*-hexadecane must have occurred.

### Proposed biochemical pathway for the synthesis of monorhamnolipids

**Figure 5:**
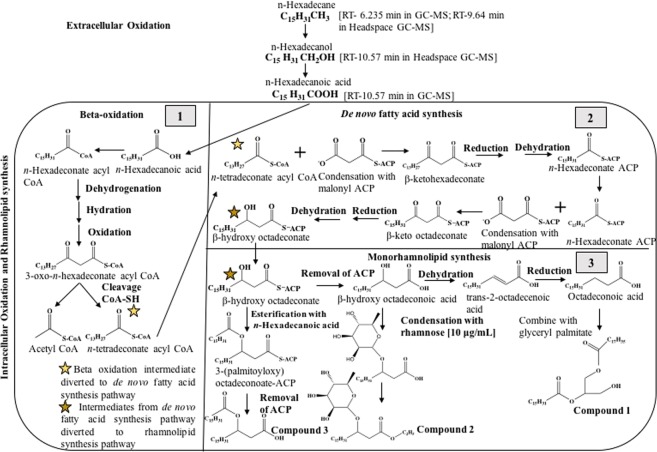
Proposed *de novo* synthetic pathway of monorhamnolipids in *Pseudomonas guguanensis*.

*n*-Hexadecane is the major component in the aliphatic fraction of crude oil and diesel and it has been used as a model molecule to study the degradation of aliphatics^32^. In the current study, we have attempted to propose a pathway to understand the mechanism of synthesis of 3 compounds produced as emulsifier by *P*. *guguanensis* with the available information on: (1) the raw materials and (2) the end products (as pictured in Fig. 5). The pathway of the production of rhamnolipid could be initiated with oxidation of the terminal methyl group of *n-*hexadecane [Headspace GC-RT: 9.64 min] to render *n*-hexadecan-1-ol, a primary alcohol as observed from the GC-MS [Headspace GC-RT: 14.73 min] and similar reaction was reported in *Pseudomonas aeruginosa* previously^33,34^. The formation of a peak at m/z 211 may be due to loss of HCHO [M-30] from 1-hexadecanol. The compound is further oxidized to *n*-hexadecanoic acid/palmitic acid [GC-RT: 10.57 min], which is reported earlier by a few researchers^33,35^. The authors indicate that these oxidative reactions are extracellular, which are mainly catalyzed by alkane hydroxylase and hexdecan-1-ol dehydrogenase before bacterial uptake. Generally the acid thus formed is taken up across the outer bacterial membranes and the only possible reaction is conjugation with coenzyme A (CoA) thus generating *n*-hexadeconate acyl CoA [C16] which is typically directed towards β-oxidation for the synthesis of acetyl CoA [C2] and *n*-tetradeconate acyl CoA [C14]. It has been reported using stable isotope tracing and gene expression assays that *n*-tetradeconate acyl coA when diverted to *de novo* fatty acid synthesis, permits higher production of lipid precursors for promoting rhamnolipid yield^36^, majorly catalyzed by the enzyme, β-ketoacyl ACP (Acyl Carrier Protein) synthase, which is a FabH-like enzyme^37^. Thus the diverted *n*-tetradeconate acyl CoA is made to enter *de novo* fatty acid synthesis pathway and upon condensation with malonyl coenzyme A [C2], forms β-ketohexadeconate-ACP [C18]. β-ketohexadeconate-ACP is reduced to β-hydroxyhexadeconate which on dehydration forms trans-2-hexadecenoyl ACP. This is converted to *n*-hexdeconate ACP which once again condenses with malonyl ACP to form β-keto octadeconate [C18] which on hydrogenation is converted into β-hydroxyoctadeconate. The product forms the precursor for rhamnolipid synthesis adopting three different routes to form compounds **1**, **2** and **3** which are isolated in the present study as bioemulsifiers.

After removal of ACP, β-hydroxyoctadeconate is converted to β-hydroxyoctadeconoic acid [a Hydroxy Fatty Acid (HAA)], which undergoes dehydration to form trans-2-octadecenoic acid which is reduced to form octadecanoic acid. The only possibility of octadecanoic acid to yield compound **1** is by esterification with glyceryl palmitate thus forming 3 hydroxy 2-palmitoxy propylstearates with a molecular formula of C_37_H_72_O_5_ [chemical structure exhaustively described by [GC-MS, FT-IR, ^1^H and ^13^C NMR] and there can be evidently no other alternate pathways. In general, it is well known that glyceryl palmitate, a triacylglycerol (TAG) is present as an important constituent in prokaryotic cell membranes and serves physiological functions especially during starvation periods and is a degradation product of *n*-hexadecane. Formation of compound **2** is initiated when β-hydroxyoctadeconoic acid condenses with a rhamnose sugar to form a monorhamnolipid containing octadecanoate [C_24_H_48_O_7_] [rhamnose content 10 μg/mL estimated using orcinol assay [datum not shown] on a MULTISKAN spectrum microplate reader, Thermo Scientific, USA]. However the proposed chemical structure for compound **2** with an added ethyl group could be because of the extraction procedures those involved the usage of ethyl acetate solvent thus forming ethyl esters of octadecenoate with molecular formula of C_25_H_50_O_7_ [GC-MS, FT-IR, ^1^H and ^13^C NMR]. Finally, β-hydroxy octadeconate also undergoes esterification with free fatty acids such as hexadecanoic acid in the cell to form 3-(palmitoyloxy) octadecenoate ACP within the cell and after the removal of ACP forms **3**-(palmitoyloxy) octadecanoate [C_34_H_66_O_4_]. However, 3-(palmitoyloxy) octadecenoate is converted to compound **3**, methyl-3-(palmitoyloxy) octadecenoate [C_35_H_68_O_4_] [GC-MS, FT-IR, ^1^H and ^13^C NMR], which again could have happened during the process of extraction, drying or during a GC-MS analysis where there are numerous possibilities of esterification of free fatty acids into methyl-esters. Hence the most probable route for the synthesis of bioemulsifiers is proposed by us, which may not happen otherwise opting any other routes. The proposed route is clearly dictated by the complete chemical structures of (1) the raw materials and (2) the end-products.

In conclusion, we mention that we have isolated a high molecular weight monorhamnolipid which is attached to esters of palmitic and stearic, which is an unusual type of a bioemulsifier. *P*. *guguanensis* is a continuous producer of this rhamnolipid when the organism is fed with only *n*-hexadecane as its sole carbon source. The secreted emulsifiers are capable of degrading hydrocarbons, with most preference to *n-*hexadecane. The used-up *n*-hexadecane is biotransformed to prepare its own emulsifiers [as seen in GC-MS fragmentation patterns] which in return is utilized to degrade *n*-alkanes [kerosene, diesel, *n*-hexadecane] thus creating a circular “biosynthesis-degradation-uptake-utilization” pathway. Currently rhamnolipids for commercial use is produced only from *Pseudomonas aeruginosa* and the price is soaring and therefore search for alternate sources become imperative. Hence we suggest, based on our investigation, monorhamnolipids from *P*. *guguanensis* could also be used as a source for clearing *n*-hexadecane from aquatic environs and could be produced in considerable quantities for commercial need. Also, the bacterium, as of now is not reported for pathogenicity and hence can be seen as a viable solution for large-scale production of eco-friendly hydrocarbon dispersants.

## Supplementary information

S1,S2,S3,S4,S5,S6,S7,S8,S9
