# Supplementary material for: Structure elucidation and proposed de novo synthesis of an unusual mono-rhamnolipid by Pseudomonas guguanensis from Chennai Port area
Source: Sci Rep. 2019 Apr 12;9:5992. doi: 10.1038/s41598-019-42045-9 (PMC6461634; doi:10.1038/s41598-019-42045-9)
Supplement: Supplementary file 1 — S1,S2,S3,S4,S5,S6,S7,S8,S9 [file 41598_2019_42045_MOESM1_ESM.doc]

**S1. Isolation of bacteria from various locations across Tamil Nadu coast**

**
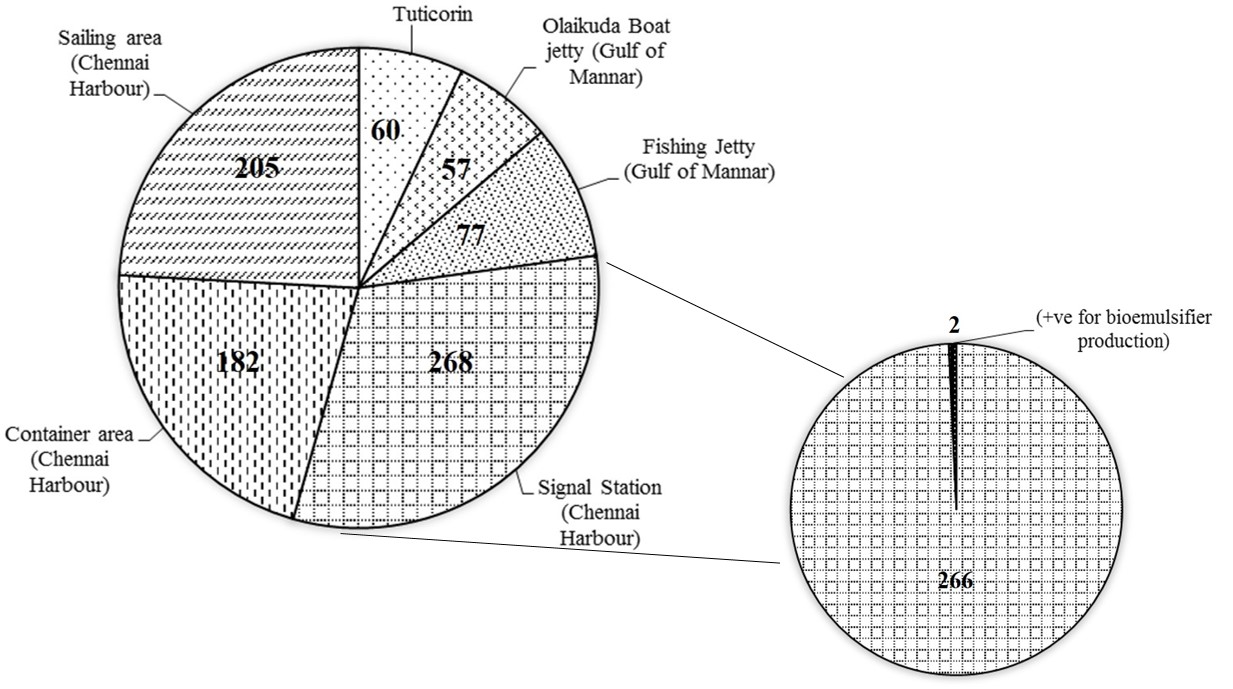
**


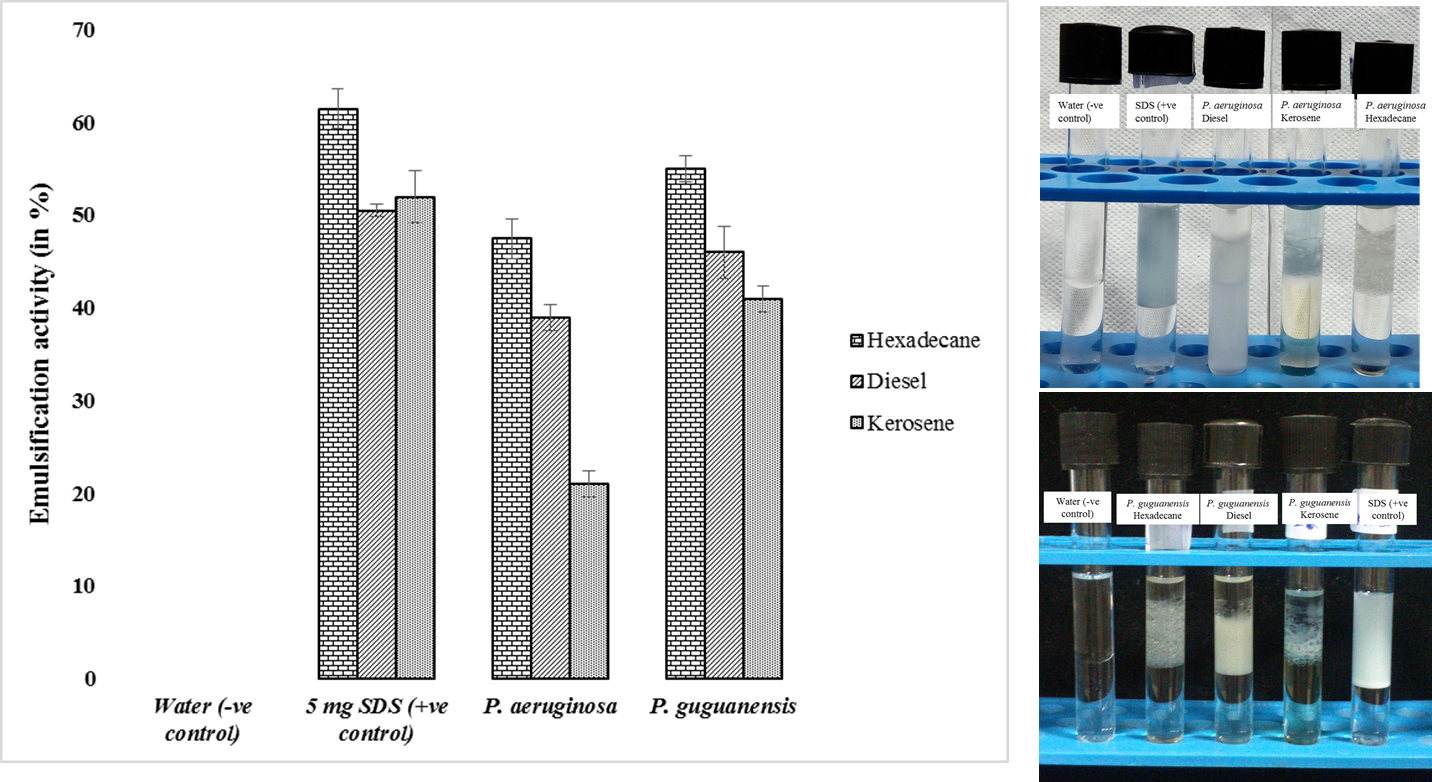
**S2. Emulsification activity of 2 mL of the spent medium [~1 OD at A_660_] of isolates A and B**

**S3. 16S rRNA gene sequences of *Pseudomonas guganensis* submitted to GenBank [Accession No:** KU302611**] (up) and its phylogenetic tree construct (down)**

GCAGTCGAGCGGATGAGGGGAGCTTGCTCCCTGATTTAGCGGCGGACGGGTGAGTAATGCCTAGGAATCTGCCTGGTAGTGGGGGATAACGTTCCGAAAGGAACGCTAATACCGCATACGTCCTACGGGAGAAAGCAGGGGACCTTCGGGCCTTGCGCTATCAGATGAGCCTAGGTCGGATTAGCTAGTTGGTGAGGTAATGGCTCACCAAGGCGACGATCCGTAACTGGTCTGAGAGGATGATCAGTCACACTGGAACTGAGACACGGTCCAGACTCCTACGGGAGGCAGCAGTGGGGAATATTGGACAATGGGCGAAAGCCTGATCCAGCCATGCCGCGTGTGTGAAGAAGGTCTTCGGATTGTAAAGCACTTTAAGTTGGGAGGAAGGGCTGCTGGTTAATACCCTGCAGTTTTGACGTTACCAACAGAATAAGCACCGGCTAACTTCGTGCCAGCAGCCGCGGTAATACGAAGGGTGCAAGCGTTAATCGGAATTACTGGGCGTAAAGCGCGCGTAGGTGGTTTTGTAAGTTGGAGGTGAAATCCCCGGGCTCAACCTGGGAACTGCCTCCAAAACTGCATGACTAGAGTACGGTAGAGGGTGGTGGAATTTCCTGTGTAGCGGTGAAATGCGTAGATATAGGAAGGAACACCAGTGGCGAAGGCGACCACCTGGACTGATACTGACACTGAGGTGCGAAAGCGTGGGGAGCAAACAGGATTAGATACCCTGGTAGTCCACGCCGTAAACGATGTCAACTAGCCGTTGGAATCCTTGAGATTTTAGTGGCGCAGCTAACGCATTAAGTTGACCGCCTGGGGAGTACGGCCGCAAGGTTAAAACTCAAATGAATTGACGGGGGCCCGCACAAGCGGTGGAGCATGTGGTTTAATTCGAAGCAACGCGAAGAACCTTACCTGGCCTTGACATGCTGAGAACTTTCCAGAGATGGATTGGTGCCTTCGGGAACTCAGACACAGGTGCTGCATGGCTGTCGTCAGCTCGTGTCGTGAGATGTTGGGTTAAGTCCCGTAACGAGCGCAACCCTTGTCCTTAGTTACCAGCACCTCGGGTGGGCACTCTAAGGAGACTGCCGGTGACAAACCGGAGGAAGGTGGGGATGACGTCAAGTCATCATGGCCCTTACGGCCAGGGCTACACACGTGCTACAATGGTCGGTACAAAGGGTTGCCAAGCCGCGAGGTGGAGCTAATCCCATAAAACCGATCGTAGTCCGGATCGCAGTCTGCAACTCGACTGCGTGAAGTCGGAATCGCTAGTAATCGTGAATCAGAATGTCACGGTGAATACGTTCCCGGGCCTTGTACACACCGCCCGTCACACCATGGGAGTGGGTTGCTCCAGAAGTAGCTAGTCTAAC


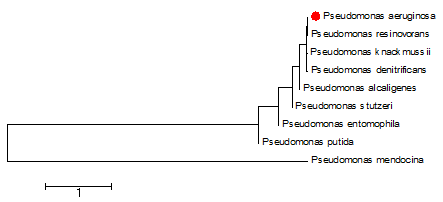

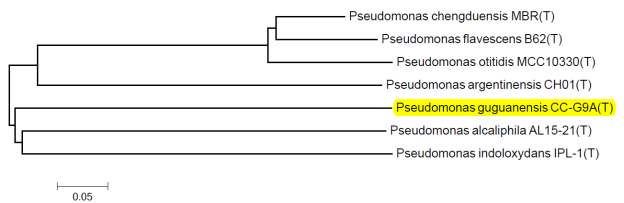

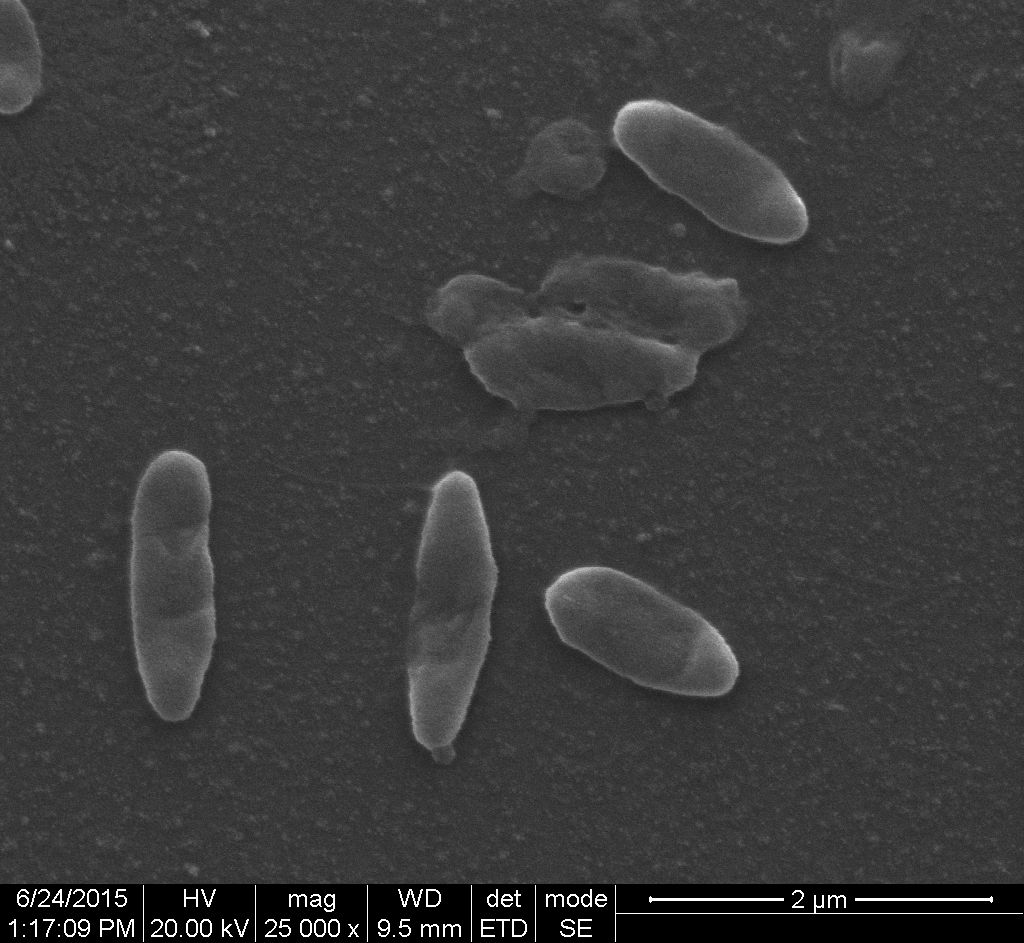


***Pseudomonas guguanensis***


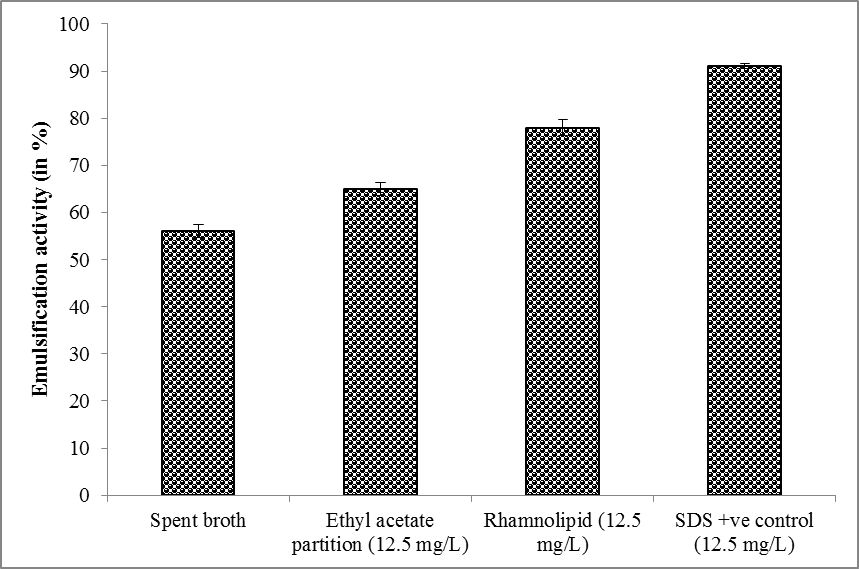
**S~~4~~. Comparison of EI*_24_* of spent broth, crude ethyl acetate partitions and rhamnolipids of *P. guguanensis***

**S5. FT-IR Spectrum of compound 1, (3 hydroxy-2-(palmytoyloxy) propyl stearate) [up] and its GC-MS fragmentation pattern [down]:**


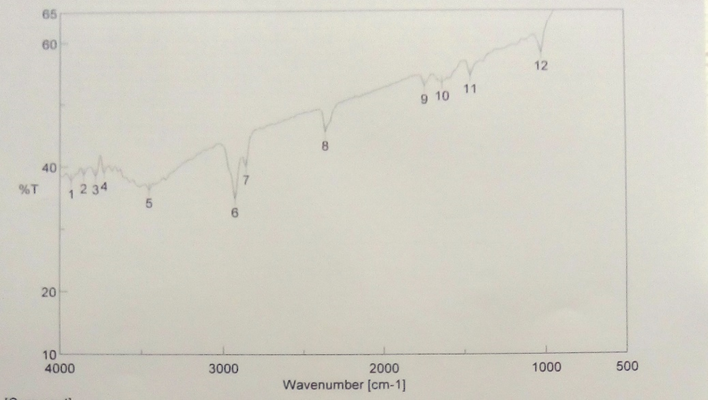


**S6. Two dimensional NMR spectroscopies compound 1 [3 hydroxy-2-(palmytoyloxy) propyl stearate]:**

**
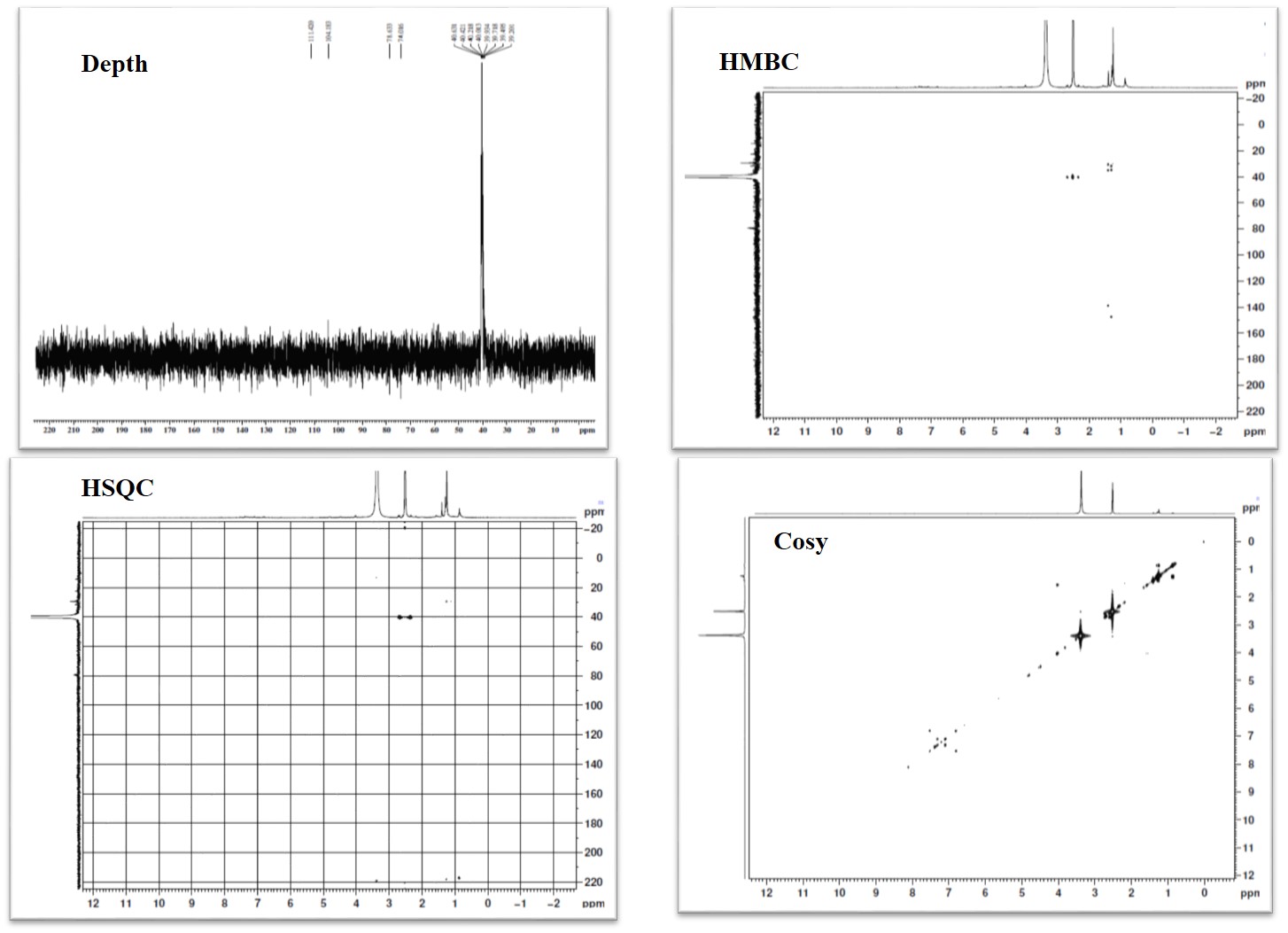
**

**S7. FT-IR Spectrum of compound 2 (ethyl 3-(((3*S,*4*S,*5*S,*6*R*)-3,4,5-trihydroxy-6-methyltetrahydro-2H-pyran-2-yl)oxy)octadecanoate) [up] and its GC-MS fragmentation pattern [down]:**


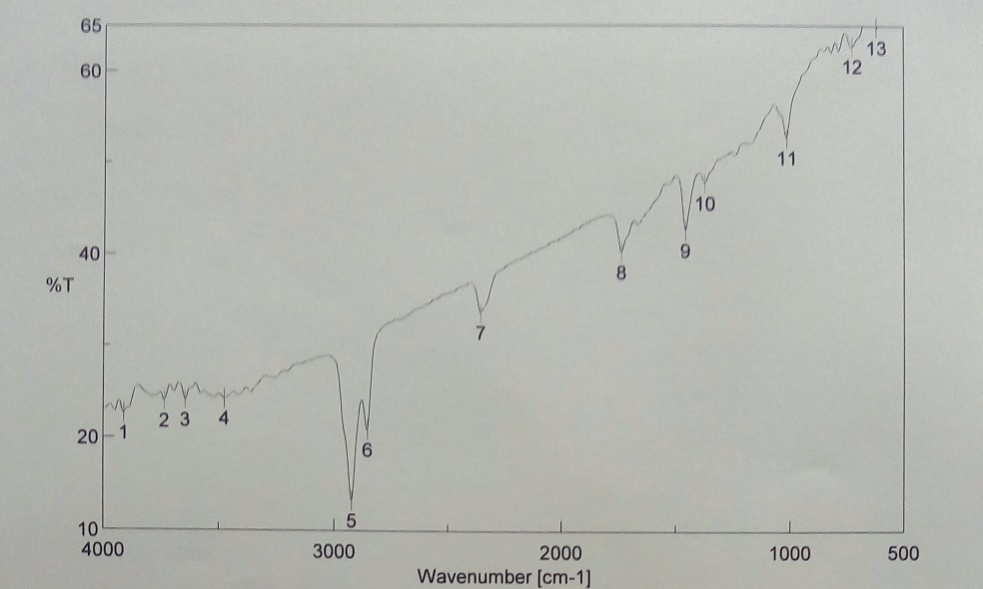


**S8. Two dimensional NMR spectroscopies of compound 2 (ethyl 3-(((3*S,*4*S,*5*S,*6*R*)-3,4,5-trihydroxy-6-methyltetrahydro-2H-pyran-2-yl)oxy)octadecanoate**
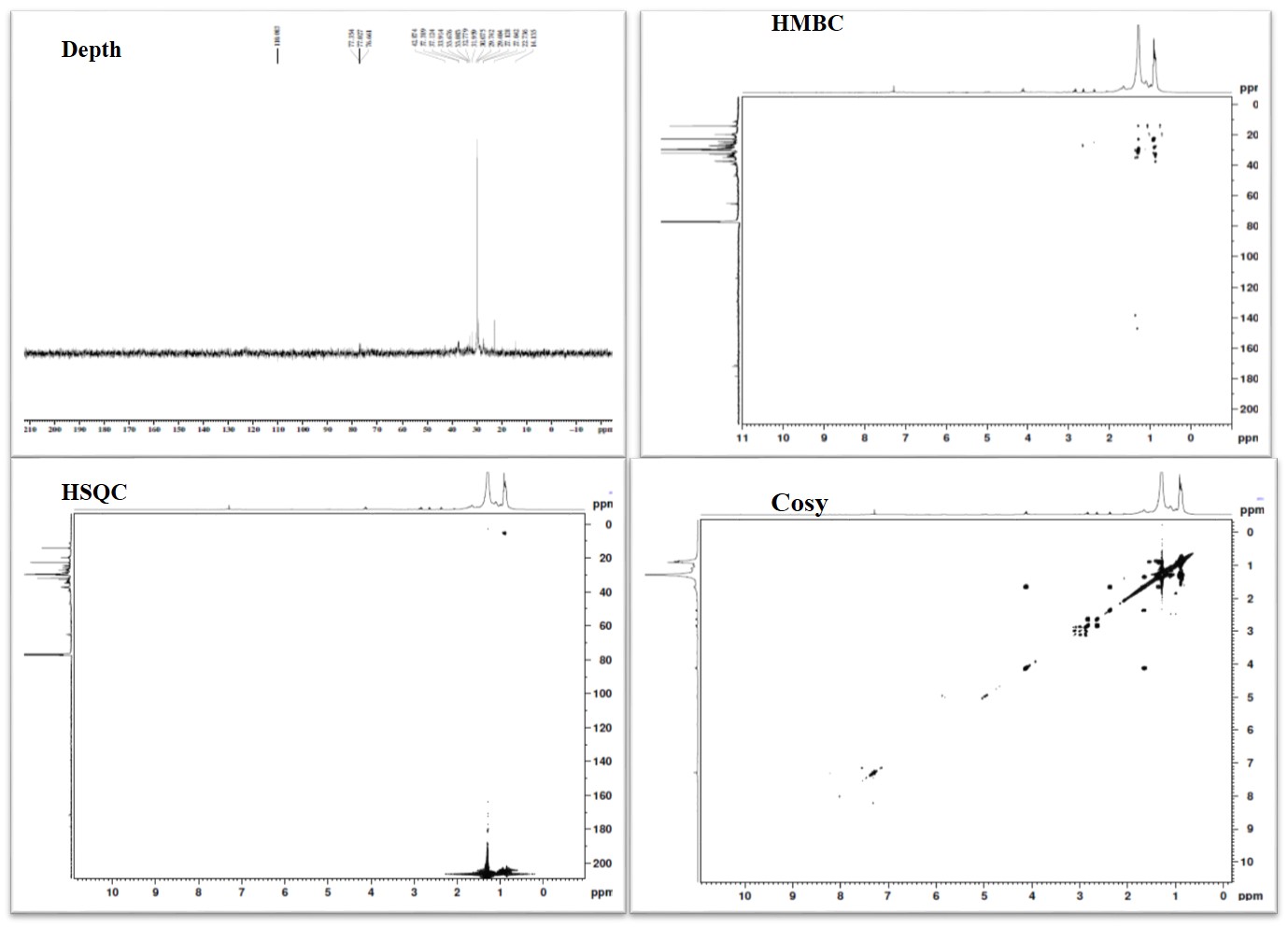
**)**

**DEPT**

**S9.**  **FT-IR Spectrum of compound 3 (methyl 3-(palmitoyloxy) octadecanoate) [up] and its corresponding two dimensional NMR spectroscopies [down]:**


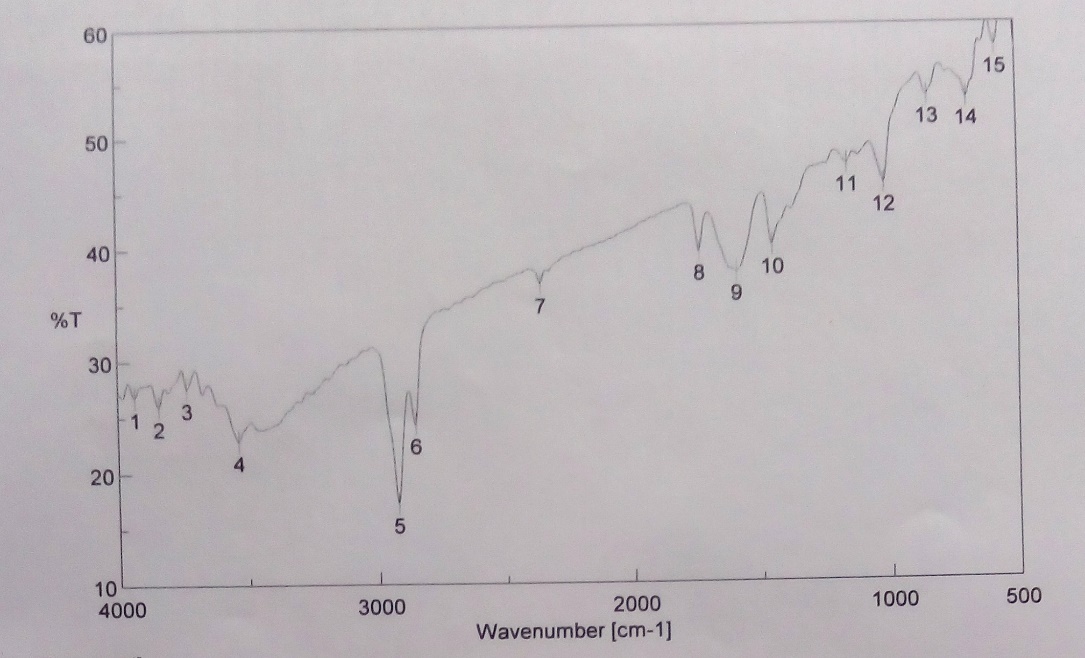

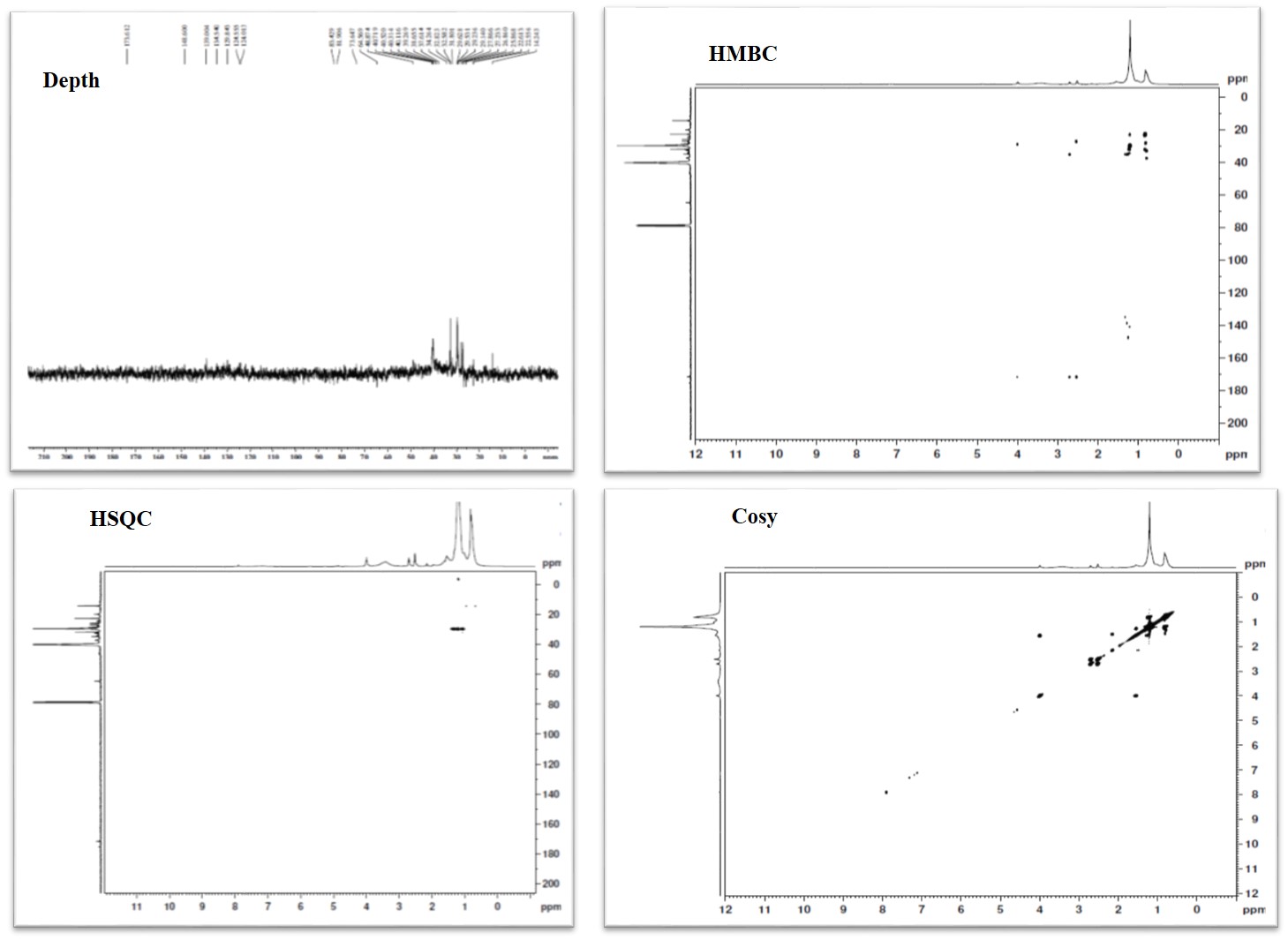


**DEPT**
